# Network toxicological insights into DEHP exposure and thyroid cancer development and progression

**DOI:** 10.3389/fonc.2025.1617202

**Published:** 2025-08-18

**Authors:** Yuhang Zhang, Qiang Wang

**Affiliations:** Department of Thyroid Surgery, Shanxi Provincial People’s Hospital, Tai Yuan, China

**Keywords:** DEHP, network toxicological, thyroid cancer, TCGA, GEO

## Abstract

This study aimed to identify markers of di-2-ethylhexyl phthalate (DEHP) exposure associated with thyroid cancer occurrence and prognosis by integrating network toxicology and molecular docking. Expression profiles and clinical information were obtained from TCGA-THCA and five GEO datasets (GSE3467, GSE3678, GSE33630, GSE53157, and GSE60542). Venn diagram analysis revealed six overlapping genes (CYP1B1, ABCC3, KRT19, CUX2, GABRB2, and TNFSF15) between the combined dataset and DEHP’s target genes. GO and KEGG enrichment analyses were conducted on these overlapping genes. Through multivariate COX regression model, it is clearly seen that CYP1B1, GABRB2 and TNFSF15 are highly expressed, and can basically be determined as candidate hub genes. Kaplan-Meier survival analysis indicated that the high-risk group had a significantly poorer prognosis (p < 0.05). Furthermore, prognostic ROC curves based on the GEO validation set demonstrated that CYP1B1, GABRB2, and TNFSF15 were significantly associated with thyroid cancer diagnosis (AUC exceeding 0.86). Finally, molecular docking was employed to visualize the interaction sites between DEHP and its target genes. In conclusion, this study provides novel targets for the prevention and treatment of thyroid cancer in the context of DEHP exposure.

## Introduction

There are numerous endocrine disruptors in the environment, including bisphenol A and phthalates, which have garnered significant attention in current research. These compounds are predominantly utilized in the manufacturing of plastics and cosmetics (1), as well as in food packaging and building materials (2). Notably, di-2-ethylhexyl phthalate (DEHP), a primary phthalate, is frequently detected in both environmental samples and human tissues (3) and is classified as potentially carcinogenic to humans (2).

The incidence of Thyroid Cancer (TC) is rising annually, with the majority of cases originating from follicular epithelial cells. These include papillary thyroid carcinoma (PTC), follicular thyroid carcinoma (FTC), poorly differentiated thyroid carcinoma (PDTC), and anaplastic thyroid carcinoma (ATC). A study has demonstrated a significant association between exposure to bisphenol A (BPA) and di(2-ethylhexyl) phthalate (DEHP) and the risk of differentiated thyroid cancer (DTC) in patients with thyroid nodules (4). Additionally, experiments conducted on rats have shown that DEHP enhances the carcinogenic effects of BPA on the thyroid gland (5).

Recently, a growing body of evidence suggests that DEHP disrupts the function of the hypothalamic-pituitary-thyroid (HPT) axis (6, 7). As a widely used plasticizer, DEHP can bioaccumulate through the food chain, leading to abnormal thyroid hormone (T3/T4) levels and oxidative stress (8). Studies have indicated that DEHP can induce thyroid hyperplasia via the arachidonic acid pathway, offering new insights into the potential link between DEHP exposure and thyroid cancer (9). Moreover, human urine tests have revealed a correlation between phthalate exposure and the incidence of papillary thyroid carcinoma (PTC), although further validation is required (10).

Most cases of highly differentiated thyroid cancer are asymptomatic and are typically detected during routine physical examinations or incidentally through diagnostic imaging. Consequently, timely targeted therapy is critical for improving patient outcomes (11). Despite growing evidence, the precise molecular networks and key driver genes linking DEHP exposure to thyroid cancer progression at a systems level remain incompletely understood. To address this knowledge gap, systems biology approaches, which integrate multi-omics data to dissect complex molecular interactions and identify core regulatory networks, have emerged as a powerful tool - their ability to capture the overall dynamics of biological systems aligns with the need to reveal the intricate links between environmental exposure (DEHP) and cancer progression. This study aims to elucidate the molecular network of thyroid cancer induced by DEHP and explore its potential association with clinical therapeutic targets. Specifically, by analyzing genomic datasets using systems biology methods, we can systematically map the molecular pathways responsive to DEHP, identify hub genes mediating thyroid cancer occurrence, and ultimately bridge the gap between environmental exposure mechanisms and the discovery of therapeutic targets.

## Materials and methods

### Data acquisition for thyroid cancer

RNA-seq data, clinical data, and survival data in FPKM format for THCA were retrieved from the TCGA database (https://gdc-hub.s3.us-east-1.amazonaws.com/download/TCGA-THCA.star_fpkm.tsv.gz; Version 05-09-2024, n=580). Differential expression analysis was conducted using R 4.2.1 to generate volcano plots and heatmaps. Additionally, datasets GSE3467 (n=18), GSE3678 (n=14), GSE33630 (n=105), GSE53157 (n=27), and GSE60542 (n=92) were obtained from the GEO database. Differential expression analysis was performed using Xiantao Academic software using DESeq2[1.36.0] and edgeR [3.38.2] package. In the GEO merged dataset, DEGs were identified based on the criteria of adj.P.Val < 0.05 and |log2(FC)| > 1, resulting in the generation of volcano plots and heatmaps. However, in the TCGA dataset, |log2(FC)| > 0.2 was used, resulting in a smaller number of differentially expressed genes in the TCGA dataset. Therefore, after multiple attempts, this threshold was chosen.

### Data acquisition for DEHP

The targeted molecular targets of DEHP were identified through an extensive search of the Comparative Toxicogenomics Database (CTD), Bioactive database of bioactive molecules with drug-like properties (ChEMBL), and Search Tool for Interactions of Chemicals (STITCH) databases. In the STITCH database, the default query is human, and molecular targets with medium confidence (> 0.4) are selected for filtering and screening. Select individuals from the CTD and ChEMBL database to screen for chemical-gene interactions. This target is a key site for chemical substances to exert biological effects. Following data integration from these sources, duplicate entries were removed to ensure data integrity.

### Venn diagram

After intersecting the differential genes identified from TCGA, GEO, and DEHP datasets, we obtained the key pathogenic genes associated with DEHP-induced thyroid cancer and constructed a Venn diagram to illustrate the overlap. Screen out the genes that simultaneously appear in the three lists of DEHP target genes, TCGA DEGs and GEO DEGs. These genes have higher research value because they are not only related to DEHP, but also have expression differences between tumor tissues and normal tissues, and also show differential expression under other experimental conditions. It might be the key gene of DEHP that affects the biological behavior of cells.

### Functional enrichment analysis

The Gene Ontology (GO) terms and the Kyoto Encyclopedia of Genes and Genomes (KEGG) pathways were analyzed using the clusterProfiler[4.4.4] package of Xiantao Academic, and significantly rich GO categories and KEGG pathways were identified. The statistical tests adopted in enrichment analysis are based on hypergeometric tests, which are typically used to evaluate the significance of GO and KEGG enrichment. The critical value of P for determining significance was set at 0.05. To correct the multiple tests, the Benjamini-Hochberg method was used to adjust the P value, thereby controlling the false detection rate (FDR). Only the GO classification and KEGG pathways with an adjusted P value less than 0.05 were considered significantly enriched.

### Screening of hub genes

In the process of screening hub genes and developing prognostic models, we first conducted a univariate Cox regression analysis on six differentially expressed genes to evaluate the impact of each gene on disease survival. Subsequently, based on the results of the univariate analysis, a multivariate Cox regression analysis was further conducted to adjust for potential confounding factors (age, sex, tumor stage) and determine independent prognostic factors. Based on the results of multivariate Cox regression analysis, we developed a prognostic model to assess the disease risk of patients. The Risk Score of this model is calculated through the following formula:

Risk Score = (CYP1B1 expression level * coefficient) + (GABRB2 expression level * coefficient) + (TNFSF15 expression level * coefficient). And a risk factor correlation map was established.

Kaplan-Meier (KM) curves associated with risk factors were generated to further investigate patient survival. When dividing the high-risk group and the low-risk group, we used the median of the risk score as the critical value. In addition, a time-varying receiver operating characteristic (ROC) curve was constructed through the timeROC package to evaluate the accuracy of survival prediction, and the results were visualized using the ggplot2 package.

### Check analysis

The ROC curve was used to evaluate the prognostic model for survival prediction in the GSE3467, GSE3678 and GSE33630 datasets.

### Molecular docking

The three-dimensional structure of DEHP (PubChem CID: 8343) was retrieved from the PubChem database. The two-dimensional structure of DEHP was softened to a three-dimensional structure through Chem3D software. Thereafter, the protein’s 3D structure was acquired from the Protein Data Bank (PDB), and its species were all human, which were de-polymerized, de-small molecule ligands, hydrogenated and de-watered using Pymol software (http://www.pymol.org/). Autodock vina program (https://vina.scripps.edu/) was used for semi-flexible docking of a small-molecule ligand to a large-molecule receptor. Semi-flexible docking experiments were conducted on the target molecules using AutoDock 1.5.7 software to evaluate their binding affinity with receptor proteins. Each molecular docking experiment was run 20 times to ensure the reliability and statistical significance of the results. Each run uses a different initial conformation to explore the possible optimal binding mode between the receptor and the ligand. The semi-empirical free energy scoring function (AutoDock 4.0 scoring function) built into AutoDock was used. This function combines factors such as van der Waals forces, electrostatic interactions, and hydrophobic effects to evaluate the binding affinity between ligands and receptors. A binding energy less than -5 kcal/mol is generally regarded as indicating a good binding ability between the ligand and the receptor. The resultant docking configurations were visualized utilizing the PYMOL software.

### Differential gene expression comparison

The key genes were compared and analyzed, and a box plot was generated.

### Immunohistochemical sections of differential genes

The key genes were retrieved from the Human Protein Atlas (HPA) database, and the corresponding pathological sections were acquired.

## Result

### Acquisition of differential genes

**Figure 1:**
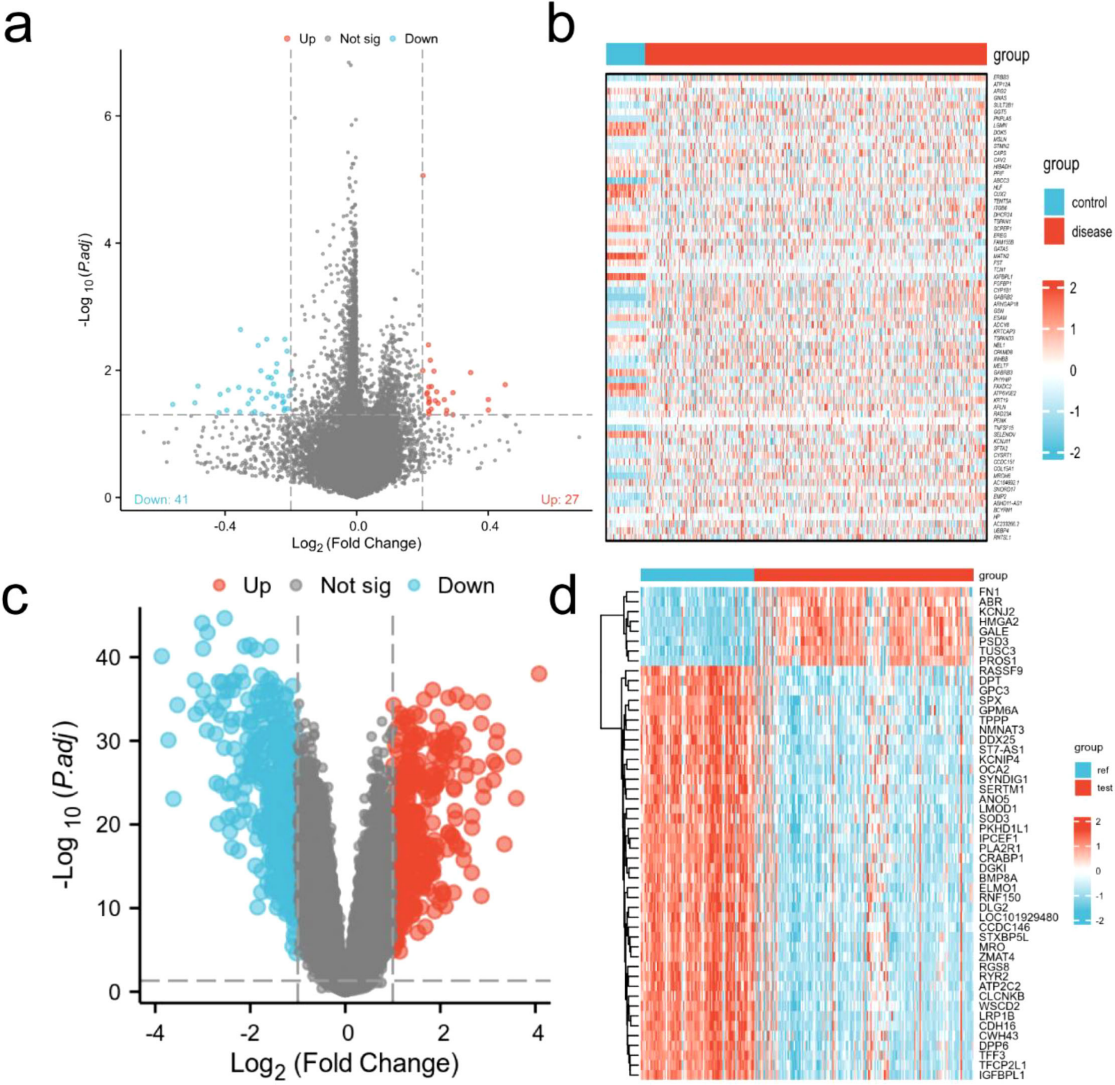
Visualization of differential genes. **(a)** Volcanic map of TCGA-THCA (adj.P.Val < 0.05 and |log2(FC)| > 0.2); **(b)** Heat map of TCGA-THCA; **(c)** Volcano map from GEO database (adj.P.Val < 0.05 and |log2(FC)| > 1); **(d)** Heat map of GEO database.

A total of 68 differentially expressed genes were identified from the TCGA-THCA database, with 41 down-regulated genes and 27 up-regulated genes. while 716 differentially expressed genes were retrieved from the GEO database, 371 genes were downregulated and 345 genes were up-regulated. The volcano plots and heatmaps for both the TCGA and GEO datasets are presented in **Figure 1**.

### Intersection of target genes

**Figure 2:**
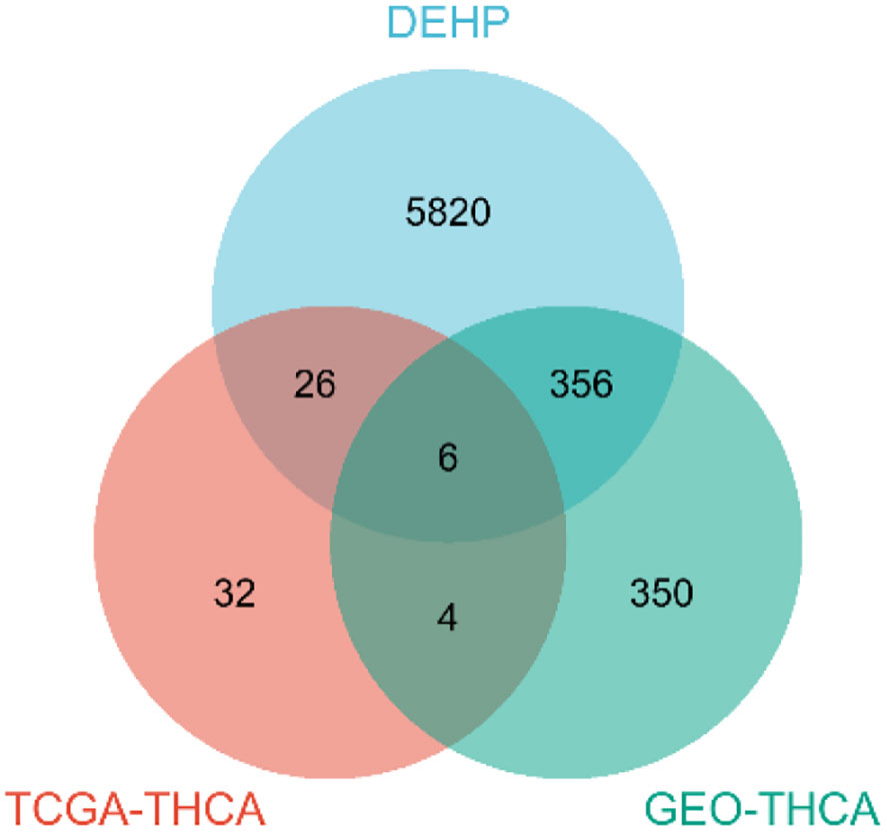
Venn diagram.

Initially, a comprehensive search identified a total of 6,209 protein targets that were associated with DEHP. After intersecting the target molecules in the DEHP database with differential genes from the TCGA and GEO databases, six target genes were identified: CYP1B1, ABCC3, KRT19, CUX2, GABRB2, and TNFSF15 (**Figure 2**).

### Functional enrichment analysis

**Figure 3:**
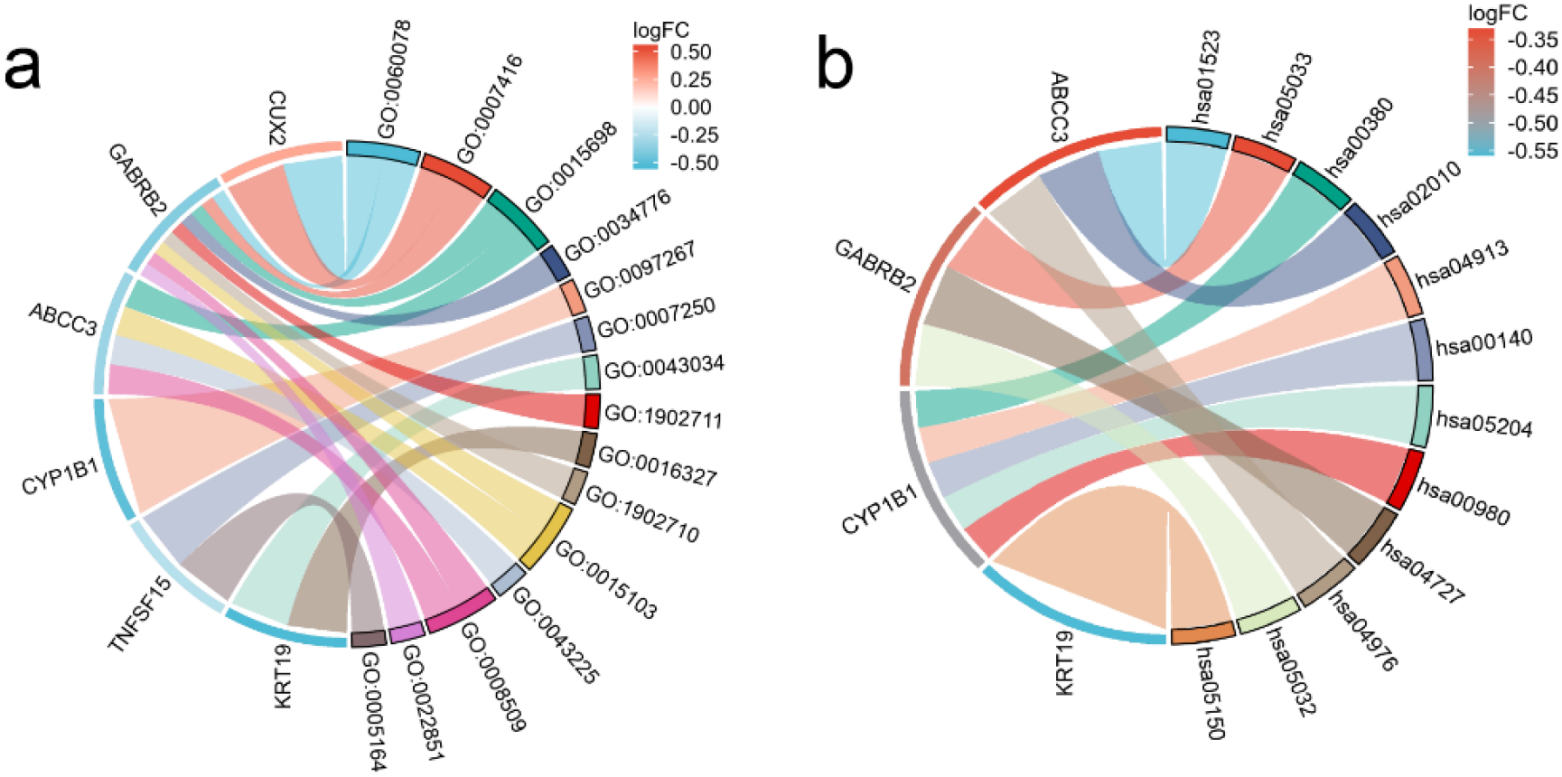
String diagram of target gene and enrichment pathway. **(a)** GO enrichment analysis; **(b)** KEGG enrichment analysis.

After performing GO and KEGG enrichment analysis on the six differentially expressed genes, it was found that GO Biological Process (BP) terms were primarily enriched in specific categories, such as regulation of postsynaptic membrane potential, synapse assembly and inorganic anion transport (**Figure 3a**). For GO Cellular Component (CC), the enrichment results are also presented, such as costamere, GABA-A receptor complex, apicolateral plasma membrane. In the Molecular Function (MF) category, the primary enrichment was observed in the pathways related to inorganic anion transmembrane transporter activity and ATPase-coupled inorganic anion transmembrane transporter activity. KEGG pathway analysis revealed a concentration in particular pathways, such as Antifolate resistance, Nicotine addiction and Tryptophan metabolism (**Figure 3b**). **Table 1** provides a detailed description of the specific pathways and their respective proportions within each enrichment category.

**Table 1:** Functional enrichment analysis.

Ontology	ID	Description	pvalue	FDR
BP	GO:0060078	regulation of postsynaptic membrane potential	7.10E-04	3.44E-02
BP	GO:0007416	synapse assembly	1.33E-03	3.44E-02
BP	GO:0015698	inorganic anion transport	1.36E-03	3.44E-02
BP	GO:0034776	response to histamine	3.19E-03	3.44E-02
BP	GO:0097267	omega-hydroxylase P450 pathway	3.19E-03	3.44E-02
BP	GO:0007250	activation of NF-kappaB-inducing kinase activity	5.73E-03	3.44E-02
CC	GO:0043034	costamere	4.89E-03	2.70E-02
CC	GO:1902711	GABA-A receptor complex	5.80E-03	2.70E-02
CC	GO:0016327	apicolateral plasma membrane	6.11E-03	2.70E-02
CC	GO:1902710	GABA receptor complex	6.41E-03	2.70E-02
MF	GO:0015103	inorganic anion transmembrane transporter activity	9.81E-04	4.29E-02
MF	GO:0043225	ATPase-coupled inorganic anion transmembrane transporter activity	3.26E-03	4.29E-02
MF	GO:0008509	anion transmembrane transporter activity	4.18E-03	4.29E-02
MF	GO:0022851	GABA-gated chloride ion channel activity	4.23E-03	4.29E-02
MF	GO:0005164	tumor necrosis factor receptor binding	1.01E-02	4.43E-02
KEGG	hsa01523	Antifolate resistance	1.82E-02	3.57E-02
KEGG	hsa05033	Nicotine addiction	2.43E-02	3.57E-02
KEGG	hsa00380	Tryptophan metabolism	2.55E-02	3.57E-02
KEGG	hsa02010	ABC transporters	2.73E-02	3.57E-02
KEGG	hsa04913	Ovarian steroidogenesis	3.09E-02	3.57E-02
KEGG	hsa00140	Steroid hormone biosynthesis	3.68E-02	4.76E-02
KEGG	hsa05204	Chemical carcinogenesis - DNA adducts	4.16E-02	4.97E-02
KEGG	hsa00980	Metabolism of xenobiotics by cytochrome P450	4.69E-02	5.01E-02
0.09573648				

### Establishment of prognostic model

**Figure 4:**
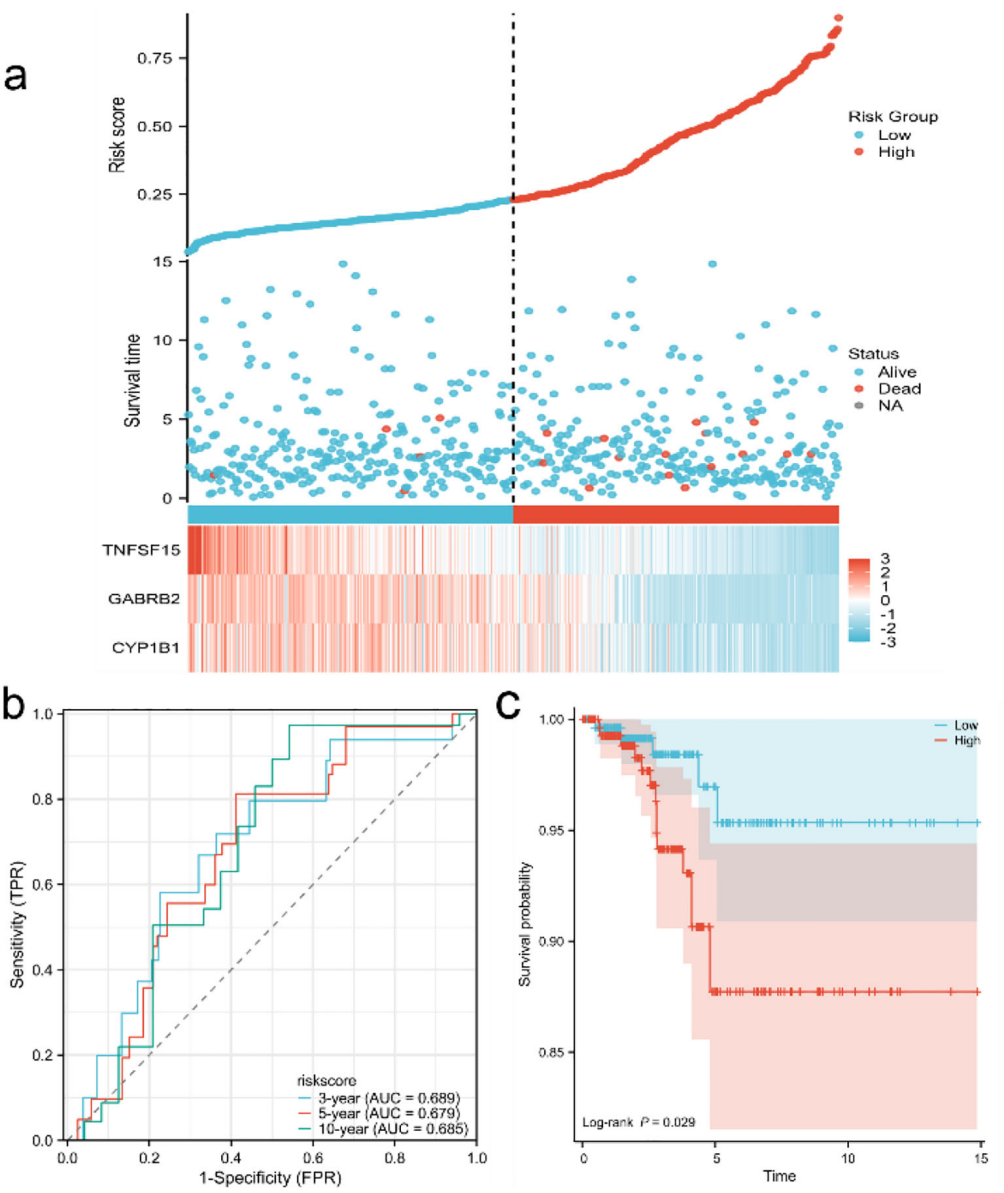
Establishment and evaluation of prognostic model **(a)** Risk factor map of risk model; **(b)** Time-dependent ROC curve of TCGA database; **(c)** KM curve of high and low risk of TCGA database.

After performing COX-molecular regression analysis on the six differentially expressed genes (**Table 2**), it was revealed that CYP1B1, GABRB2, and TNFSF15 significantly influenced disease survival and risk stratification (**Figure 4a**). **Table 3** presents. The establishment results of the multivariate COX regression model. Consequently, a prognostic model was developed. The time-dependent ROC curve analysis demonstrated that this model exhibited consistent predictive power at 3-year, 5-year, and 10-year intervals (AUC: 0.689, 0.679 and 0.685), indicating its potential clinical utility (**Figure 4b**). Additionally, survival curves generated from the TCGA database showed that patients in the high-risk group had a poorer prognosis compared to those in the low-risk group (**Figure 4c**).

**Table 2:** The establishment results of the univariate and multivariate COX regression model.

Characteristics	HR (95% CI) univariate analysis	P value univariate analysis	HR (95% CI) multivariate analysis	P value multivariate analysis
CYP1B1	0.785 (0.590 - 1.046)	0.098	0.895 (0.552 - 1.451)	0.653
ABCC3	0.754 (0.492 - 1.154)	0.193	–	–
KRT19	0.847 (0.672 - 1.068)	0.161	–	–
CUX2	1.343 (0.828 - 2.179)	0.232	–	–
GABRB2	0.705 (0.495 - 1.002)	0.051	0.876 (0.458 - 1.676)	0.690
TNFSF15	0.440 (0.182 - 1.063)	0.068	0.562 (0.227 - 1.392)	0.213

**Table 3:** The establishment results of the multivariate COX regression model.

Multi-factor model variables	Coeff
Intercept	1.490080476
CYP1B1	-0.110693351
GABRB2	-0.131957651
TNFSF15	-0.576752188

### Validation of target genes

**Figure 5:**
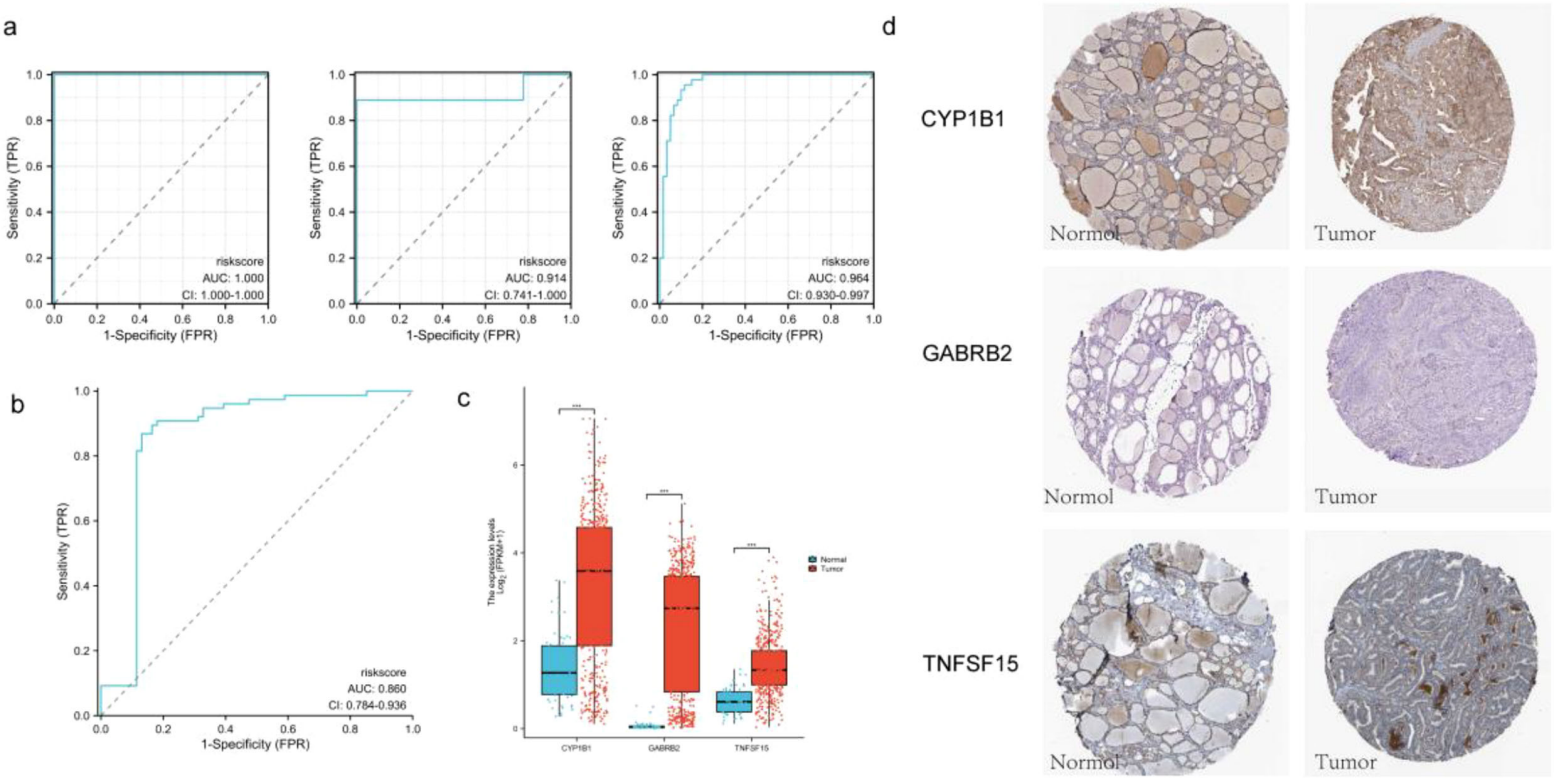
**(a)** The ROC curves of the validation set are GSE3467, GSE3678 and GSE3363 respectively; **(b)** The ROC curve of the combined validation set. **(c)** Grouping expression box maps of three key genes; **(d)** IHC pathological sections of three key genes (DAB staining; 40x; CYP1B1-normol HPA026863, CYP1B1-tumor HPA026863; GABRB2-normol HPA067632, GABRB2-tumor HPA067632; TNFSF15-normol HPA067632, TNFSF15-tumor HPA067632).

The model was validated using five datasets (GSE3467, GSE3678 and GSE3363) from the GEO database. The areas of the ROC curves of a single dataset were divided into 100%, 91.4% and 96.4% (**Figure 5a**). The area under the ROC curve reached 86%, demonstrating the high reliability of the model (**Figure 5b**). Furthermore, by analyzing key genes, we observed that their expression levels were significantly higher in the cancer group compared to the normal group (**Figure 5c**) and p was significantly less than 0.05. Immunohistochemistry (IHC) images of these three key genes obtained from the HPA database provided additional evidence supporting their differential expression (**Figure 5d**).

### Molecular docking

**Figure 6:**
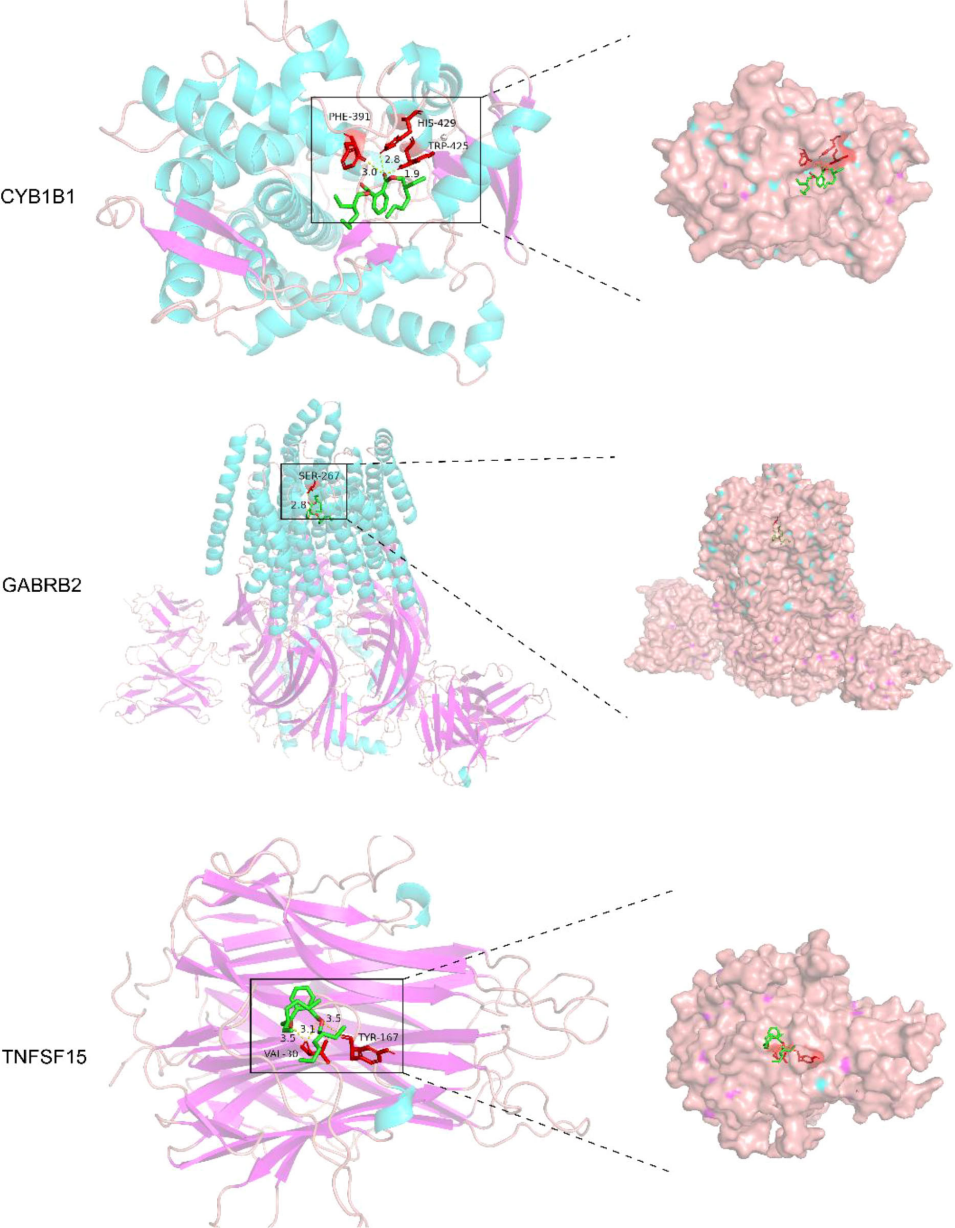
The molecular docking models of DEHP with three key proteins, generated using AutoDock 1.5.7 for semi-flexible docking, were visualized utilizing PyMOL.

Molecular docking studies were conducted to investigate the interactions between DEHP and the proteins CYP1B1 (pdb_00003pm0), GABRB2 (pdb_00006x3t), and TNFSF15 (pdb_00002re9). **Table 4** shows the details of the docking between DEHP and the corresponding target. **Figure 6** shows the molecular schematic diagram and the surface model respectively through the left and right panels. The black box in the left image focuses on the specific docking site, which is the active region where the ligand binds to the protein receptor. Here, the amino acid residues of the ligand and receptor interact through hydrogen bonds, and other means to form a fitting binding pocket. The 3D surface model on the right further presents the three-dimensional structure of the combined pocket, clearly demonstrating the complementarity between its shape and ligands, as well as how the surface charge distribution affects their combination, providing an intuitive basis for understanding the intermolecular interaction mechanism.

**Table 4:** Molecular docking binding force.

Stem	Types of interactions	Protein affinity (kcal/mol)	Amino acid residues bonded by hydrogen bonds	Gridbox (center: x, y, z)
CYP1B1	Hydrogen bonds	-6.5	PHE-391 HIS-429 TRP-425	-16.978, 17.636, -11.956
GABRB2	Hydrogen bonds	-5.8	SER-267	149.111, 133.973, 134.406
TNFSF15	Hydrogen bonds	-5.3	VAL-30 TYR-167	-5.253, 27.777, -14.643

## Discussion

This study investigates the network toxicological mechanisms underlying thyroid cancer induced by DEHP. CYP1B1, GABRB2, and TNFSF15 have been identified as significant target genes that can collectively predict the prognosis of thyroid cancer and possess high clinical value. Furthermore, these genes may play a crucial role in targeted therapy for thyroid cancer.

In human epidemiological studies (12, 13) and in animal and *in vitro* experiments (14–17), phthalate plasticizers, particularly DEHP, have been shown to affect the hypothalamic-pituitary-thyroid (HPT) axis (18) and disrupt thyroid hormone homeostasis. Such interferences may potentially account for the recent high incidence of giant thyroid tumors (19). Studies have demonstrated a significant association between thyroid-related hormones and the risk of thyroid cancer (TC) (20). Thyroid hormones serve as mediators in tumor growth, proliferation, and progression (21, 22). Our findings build on this foundation by linking DEHP exposure to specific molecular perturbations. Based on this, our research links DEHP exposure to specific molecular perturbations, using this to explain the specific molecular mechanisms and pathways by which DEHP mediates the occurrence of thyroid cancer.

For instance, several observational studies reporting negative correlations between DEHP metabolites and thyroid hormones (T3, T4) in general populations, pregnant women, and children (23) align with our KEGG enrichment in hormone metabolism pathways, where CYP1B1-known to metabolize xenobiotics—may modulate thyroid hormone bioavailability by interacting with T4-converting enzymes.​ CYP1B1-mediated xenobiotic metabolism, creating a feedback loop that amplifies hormonal disruption and genetic instability.​We indicates CYP1B1, a cytochrome P450 enzyme, may influence this process by metabolizing thyroid hormones, thereby altering their bioavailability and signaling potency. Additionally, thyroid hormones’ involvement in cell transformation, tumorigenesis, and metastasis (22) aligns with CYP1B1’s known role in mediating epithelial-mesenchymal transition (EMT) (24), a critical step in metastasis, creating a direct connection between hormonal signaling and our identified gene.​ Moreover, elevated free thyroxine (FT4) levels and reduced free triiodothyronine (FT3) levels associated with increased TC risk (25), and the proposed (FT4/FT3) ratio as a risk indicator (26), could be linked to CYP1B1’s function. As CYP1B1 is involved in xenobiotic metabolism, its dysregulation by DEHP may disrupt T4 to T3 conversion, contributing to the imbalanced FT4/FT3 ratio observed in malignant cases.​

Additionally, Ye et al. (27) found that DEHP-induced oxidative stress and ROS elevation, which activate the Ras/Akt/TRHr pathway and reduce TSH levels, provide a mechanistic link to our target genes. Since GABRB2 downregulation inhibits PI3K/AKT activation (28), DEHP-induced dysregulation of GABRB2 may synergize with T3 receptor mutations to amplify PI3K signaling, accelerating tumor development. The T3 receptors interaction with its promoter to regulate fibroblast growth factor expression in thyroid cells, and the finding that T3 receptor mutations enhance phosphatidylinositol 3-kinase signaling leading to thyroid tumors (29), directly intersect with our analysis of GABRB2. GABRB2, whose downregulation disinhibits PI3K/AKT signaling (28), may synergize with DEHP-activated Ras/Akt pathways to exacerbate TSH suppression, creating a pro-proliferative environment.

Histological changes from DEHP exposure-thyroid follicle hypertrophy, proliferation, and inflammatory infiltration (30)-are mirrored in our GO enrichment of cell cycle and inflammatory response terms, with TNFSF15 potentially driving this infiltration by recruiting pro-tumorigenic immune cells, as supported by its enrichment in TNF superfamily pathways. Given TNFSF15’s capacity to recruit pro-tumorigenic immune cells, it may contribute to the formation of an inflammatory microenvironment that enhances TSH-induced cell proliferation and the accumulation of mutations (31, 32). Demircioglu et al. (33) reported that elevated preoperative TSH levels are associated with extrathyroidal extension and central lymph node metastasis, which aligns with the role of TNFSF15 in inflammatory signaling, as members of the TNF superfamily are frequently involved in mediating tissue invasion. The enhancement of immune function through TSH suppression therapy (34) may also involve TNFSF15, as its regulation could help mitigate inflammatory infiltration.

This study demonstrated that the prognostic model constructed based on target genes exhibits certain advantages, and integrated analysis can offer greater support for the treatment and management of thyroid cancer. This study has certain limitations. All data utilized in this paper were derived from databases and have not been validated through cellular or animal experiments. Future research will include *in vitro* validation using thyroid cell lines exposed to DEHP, and further investigation of the expression levels of CYP1B1, GABRB2 and TNFSF15 through molecular dynamics simulation and molecular interaction, as well as assessment of their functional impacts on cell proliferation, migration and apoptosis.

## Conclusion

Through computational network toxicology analysis, this study identifies CYP1B1, GABRB2, and TNFSF15 as potential molecular targets mediating DEHP’s effects in thyroid cancer. These in silico findings provide novel insights into DEHP-associated thyroid carcinogenesis and suggest promising candidates for targeted therapy and prevention. Future experimental validation is essential to confirm the therapeutic relevance of these predicted targets.

## Data Availability

The original contributions presented in the study are included in the article/supplementary material. Further inquiries can be directed to the corresponding author.
